# The Utility of a Smartphone-Based Retinal Imaging Device as a Screening Tool in an Outpatient Clinic Setting: Protocol for an Observational Study

**DOI:** 10.2196/52650

**Published:** 2025-03-25

**Authors:** Ajay Mittal, Victor Sanchez, Navjot Singh Azad, Yaroslav Zuyev, Rafael Robles, Mark Sherwood

**Affiliations:** 1 University of Florida College of Medicine Gainesville, FL United States; 2 Edward Via College of Osteopathic Medicine - Louisiana Monroe, LA United States; 3 Bascom Palmer Eye Institute, University of Miami Miami, FL United States; 4 Virginia Commonwealth University School of Medicine Richmond, VA United States

**Keywords:** digital health, digital ophthalmoscope, ophthalmology, smartphone-based, mobile health, applications, screening tool, retinal imaging device, glaucoma, eye disease, visual problems, ophthalmoscope, ocular disease, cost-effective, mobile phone

## Abstract

**Background:**

Glaucoma, a disease leading to the degeneration of retinal ganglion cells, results in changes to the optic nerve head that are often diagnosed late when visual problems arise. With the prevalence of glaucoma surpassing 76 million adults worldwide and with glaucoma being the leading cause of irreversible blindness in the world, the early detection and management of glaucoma is imperative. Digital ophthalmoscopes, such as the D-EYE (D-EYE, Srl), have emerged as a technology that uses smartphone cameras with an attachment on the lens to visualize the retina and optic nerve head without the need for dilation. The purpose of this pilot study is to examine the acceptability and feasibility of a D-EYE digital ophthalmoscope to screen for ocular pathology involving the optic nerve, particularly glaucoma.

**Objective:**

This study aimed to demonstrate the effect of a smartphone-based ophthalmoscope as a potential vision screening tool for optic nerve head pathology in participants enrolled in this study. The first specific aim was to determine the ability of the D-EYE smartphone ophthalmoscope to gather high-quality imaging to be used for grading the fundus into low- and high-risk categories for eye pathology. The second specific aim was to determine the difference in the quality of data capture between still retinal images and 30-second retinal video recordings produced by D-EYE smartphone ophthalmoscopes.

**Methods:**

This observational pilot study enrolled 110 patients receiving routine eye care at the University of Florida Health from February 2019 to February 2022 to assess the use of the D-EYE device in capturing still images and 30-second videos of the bilateral retina and optic nerves of each participant. Study participants completed a survey to gather demographics and past medical history data with a particular focus on previous eye health history. Images were reviewed by 5 ophthalmology residents with interrater reliability analysis performed to assess findings.

**Results:**

Ophthalmology resident review indicated greater visualizability and clarity of the bilateral retina and optic nerves with 30-second videos of retinal imaging compared with still-image ophthalmic capture. Furthermore, an increase in visualizability and clarity allowed for a more accurate measurement of the cup-to-disc ratio, a diagnostic marker for glaucoma. In addition, the likelihood of referral of the glaucomatous and healthy sample groups to ophthalmologists indicated a greater sensitivity of digital ophthalmoscopes in being able to detect retinal abnormalities requiring early intervention and management, supporting the technology’s use as a screening tool.

**Conclusions:**

This investigation suggests that the use of smartphone-based digital ophthalmoscopes can be more effectively applied as a screening tool by capturing 30-second videos compared with still images alone. This novel assessment of an emerging technology in the field of ophthalmology may better equip further research as smartphone camera technology advances.

**International Registered Report Identifier (IRRID):**

DERR1-10.2196/52650

## Introduction

### Vision Screening and Digital Ophthalmoscopes

Vision screening is a valuable component of health maintenance as loss of vision may lead to severe impairment in everyday tasks, such as driving, ambulating, reading, and social participation [1,2]. Glaucoma, the second leading cause of permanent blindness in the United States, is the acquired loss of retinal ganglion cells within the optic nerve, resulting in the progressive loss of peripheral vision [3,4]. The 2 most common types of glaucoma in the United States are primary open-angle glaucoma and primary angle-closure glaucoma, with most patients presenting asymptomatically until the optic nerve damage is severe enough [5]. It was estimated that by the year 2020, over 3 million adults would have glaucoma in the United States with nearly half not knowing that they have the disease [6].

Existing methods of screening for glaucoma involve comprehensive eye examinations performed by ophthalmologists. Often, patients who are at a higher risk for eye pathologies, such as glaucoma, do not participate in such screening efforts, and even when diagnosed, do not attend follow-up visits for complete eye examinations to monitor the progression of the disease. This is especially true for patients in low-income socioeconomic demographics. A study examining poor longitudinal glaucoma follow-up in India found that, adjusting for age and sex, independent predictors of poor follow-up included lack of formal education, lack of escort, and belief that follow-up is less important if one has no noticeable visual changes [7]. In addition, studies at a tertiary hospital eye department found various social and demographic factors contributing to poor adherence to follow-up, as patients who are African American or Latino, or who live far from their eye care provider are at a greater risk of nonadherence to follow-up appointments [8]. Studies performed in 3 major hospital departments found that patients presenting with advanced glaucoma are more likely to come from an underprivileged area and be of lower occupational class, to have no access to transportation, and to have a lower education level [9]. Factors such as income, education, ethnicity, and lack of transportation suggest an association between low socioeconomic status and prevalence of eye disease as well as lack of follow-up for eye health appointments.

With vision loss from glaucoma being irreversible and there existing a multitude of barriers to follow-up, early detection and management of the disease is imperative and solidifies a need for easily accessible, highly sensitive diagnostic technology. In a study assessing the comparison of smartphone ophthalmoscopy with slit-lamp biomicroscopy for grading vertical cup-to-disc (CtD) ratio (VCDR) on 110 patients, smartphone ophthalmoscopy showed substantial agreement with slit-lamp examination for the estimation of the VCDR [10]. This success in the estimation of VCDR can be used for glaucoma screening in low-resource environments. When compared with the use of a traditional direct ophthalmoscope, a 2018 Nature publication found that smartphone ophthalmoscopy produced more accurate clinical descriptions of findings in a fundal examination than direct ophthalmoscopy [11]. In addition, the study suggested that the use of a smartphone-based alternative to the direct ophthalmoscope may improve the accuracy and quality of fundal examinations by nonophthalmologists [11]. Implementation of smartphone ophthalmoscope technology may potentially be an effective way to screen patients for diseases of the eye within primary care clinics. Such an intervention would address the loss of follow-up to specialty ophthalmology clinics for complete ophthalmologic examinations in low socioeconomic status patients with eye disease. Early intervention may allow for the timely, evidence-based management of eye disease and prevent late-stage morbidities such as progressive loss of vision and permanent blindness.

### Specific Aims

This study aimed to demonstrate the effect of a smartphone-based ophthalmoscope as a potential vision screening tool for optic nerve head pathology, particularly glaucoma, in participants enrolled in this study.

- Specific aim 1: to determine the ability of the D-EYE (D-EYE, Srl) smartphone ophthalmoscope to gather high-quality imaging to be used for grading the fundus into low- and high-risk categories for eye pathology
- Specific aim 2: to determine the difference in quality of data capture between still retinal images and 30-second retinal video recordings produced by D-EYE smartphone ophthalmoscopes.

## Methods

### Participants

This pilot study will use an observational research design to assess the ability of the D-EYE to capture high-quality imaging that can be interpreted by ophthalmologists. Furthermore, the study will investigate the difference in quality of imaging between still images and 30-second videos of the retina for clinical use based on ophthalmology resident feedback. Trained research assistants who are familiar with the protocol for recruitment and eligibility to participate in this study will recruit patients in coordination with medical staff at their respective clinical sites.

The sample population will consist of 110 adults between the ages of 18 and 99 years (N=110). University of Florida (UF) Health Eye Center is the designated site where this study will be conducted.

Criteria to participate in this study include being a patient currently seen at the UF Health Eye Clinic and being between the ages of 18 and 99 years. The study will exclude patients matching the criteria such as loss of vision, blind patients, patients who require urgent procedures or are otherwise deemed unstable by medical staff, and patients with photosensitivity that prevents prolonged exposure to bright lights.

Participant data will be blinded from resident graders for review, and their existing medical records will provide a reference to determine the accuracy of interpretation from the ophthalmologist’s review and offer insight as to the quality of imaging and preferred modality based on the reported interpretability and accuracy of the 5 resident reviewers.

### Recruitment

Participants will be recruited in the study under the title, “The Utility of a Smartphone-Based Retinal Imaging Device as a Screening Tool in an Outpatient Clinic Settings.” After identifying eligible participants for this study, trained research assistants will approach and recruit them. If these eligible participants are interested, a trained study member will review and obtain consent directly from the participant. A standardized informed consent script will be adhered to ensure consistency in the consenting process. Participants will receive a copy of the informed consent document.

### Procedure

Research assistants, working in coordination with medical staff, will identify and determine the eligibility of patients entering the UF Health Eye Center for medical care. Consent will be obtained directly from the participants.

Before collecting the retinal imaging data, participants will complete a survey that provides pertinent demographic data, past medical history, and ophthalmology-specific questions regarding whether or not they have a diagnosed eye condition. After completion by the study participants, the paper files will be stored in a secured holding unit in accordance with the UF Institutional Review Board’s guidelines.

Next, participants will have their retinal imaging collected by trained research assistants using the D-EYE. The D-EYE is a US Food and Drug Administration (FDA)–approved smartphone ophthalmoscope that was used over the course of this study. It is capable of gathering high-quality images and videos of the retina. The eye exam will occur in a low-lit room. Bilateral eyes will be examined by the D-EYE for 25-35 seconds. This footage will then be temporarily stored in the D-EYE encrypted app, along with the patient’s medical record number and the patient’s known eye pathologies (if any). The smartphone camera attachment is compatible with the D-EYE app, a Health Insurance Portability and Accountability Act (HIPAA)–compliant smartphone app. From the D-EYE app, the images collected (both still images and 30-second videos) can be uploaded to a UF-affiliated, secured Dropbox (Dropbox Inc.) that has been approved for data storage. The D-EYE smartphone app requires either a 4-digit password login, fingerprint scan, or face ID to access. Video and image recordings will not be stored on the device capturing the images but will be housed on the HIPAA-compliant UF Dropbox account.

The UF Dropbox cloud-hosting service will only be accessible to study personnel and resident ophthalmologists who are required to view the retinal imaging. Access to the UF Dropbox requires logging into the UF Health VPN and accessing the site through a password combination containing uppercase letters, lowercase letters, numbers, and punctuation marks.

The footage taken by the D-EYE will be reported to UF Ophthalmology residents through Dropbox. Five residents will then grade each fundus image based on (1) the clarity of the footage on a scale of 1-10 (still image vs 30-second videos), (2) whether the residents would refer the participant to an ophthalmologist for eye care, (3) whether to categorize the sample into the “healthy” or “unhealthy or needs care” category, and (4) if there are any optic neuropathies (glaucoma) present, stratifying the findings as either low, moderate, or high risk. Their responses will be cross-referenced with the patient’s known eye history and the responses will be categorized as either hits, type I misses (resident lists as unhealthy but history suggests the eye is healthy), or type II misses (resident lists healthy but history suggests the eye is unhealthy).

### Outcomes Measures

Certain listed measures will be collected for all participants. All data will be stored through a secured, university-affiliated Dropbox account approved by the Institutional Review Board, a HIPAA-compliant and university-supported app used for data capture and storage.

#### Primary Outcomes

##### Image Quality

The clarity of both the still images and 30-second videos will be rated on a scale of 1-10. Accuracy in determining CtD measurement will be assessed between the still imaging and 30-second videos with a determination of any differences in quality and measurements of data between the still images and 30-second videos.

##### Categorization

Based on the imaging results, the ability of residents to categorize participants’ retinal imaging into either broad “healthy” or “unhealthy or needs care” groups.

##### Likelihood for Referral

Based on the imaging results, the residents will assess whether or not they would refer participants to an ophthalmologist for further specialized eye care.

##### Stratification

If there are any detectable optic neuropathies present, they will be stratified into either low-, moderate-, or high-risk groups based on the extent of the disease.

#### Secondary Outcomes

##### Interpretability

Assessment of eye health will be grouped into either hits, type I misses (resident lists findings as unhealthy but history suggests the eye is healthy), or type II misses (resident lists findings as healthy but history suggests the eye is unhealthy).

##### Interrater Agreeability

Assessment of the level of agreement between independent resident ophthalmology graders regarding the image quality, categorization, likelihood for referral, and stratification of retinal images (still images and 30-second videos) captured from the D-EYE camera attachment.

### Statistical Analysis

The information collected from the study consists of categorical data, distinguishing between accuracy and inaccuracy. The authors will be performing an interrater reliability test using the Cohen coefficient for each of the ophthalmology residents making a diagnosis. This will provide the authors with a level of agreement. All tests will be 2-sided and *P* values <.05 will be considered statistically significant.

To test the first aim, the authors will have 5 ophthalmology residents grade D-EYE images on a scale of 1-10 for clarity for the use of low-risk and high-risk stratification. Low-risk and high-risk stratification will be judged based on the likelihood that the resident will refer the patient to a specialist. Their grades will then be averaged, the SD will be calculated, and interrater reliability will be determined. Furthermore, the authors will test the extent to which clarity can be used for making a diagnosis. They will ask the residents to make a diagnosis and compare their judgments to existing patient history. These results will be stratified into hits, type 1, or type 2 misses. These will be used to calculate proportional variability (PV; PV+ and PV) values. Interrater reliability will also be calculated.

### Ethical Considerations

This study, which includes human participant research, was approved by the Institutional Review Board of the Florida Department of Health (UFIRB201801242). The informed consent forms used in this study provide participants with a description of the study, its qualitative and quantitative measures, potential risks and discomforts, and explicitly ask the participants for a voluntary agreement to participate and allow their data to be collected. To maintain privacy and confidentiality protection, study data are de-identified as all participants are assigned a numerical code. This code follows the format of OC001. Anonymity of all study participants is ensured. No compensation of any sort is offered to the human participants. This is stated in the informed consent form. No identification of individual participants is present in any images of this paper or any supplemental material.

## Results

A total of 110 participants underwent retinal screening assessments using the D-EYE attachment to iPhone 7 (Apple Inc). Both still images and 30-second videos of the participants’ bilateral fundi were captured and were then compared in order to assess the clarity and quality of the imaging.

The D-EYE video footage showed the ability to achieve optic nerve head visualization with an overall proportion of 0.827 being visualized by graders. This was a notable improvement compared with still images, where graders were only able to visualize the optic nerve head with an overall proportion of 0.752. As such, video footage captured by the D-EYE suggests efficacy compared with previously established tools, and when combined with portability and cost, it may prove to be a useful clinical tool for physicians who want to assess optic nerve head health. One previous study that assessed the D-EYE showed that still imaging was possible in 74% of undilated cases [11]. This research corroborates this finding and also suggests that more consistent and better visualization could be achieved through the use of video rather than still imaging.

The intraclass correlation coefficient found when comparing grader CtD assessments was 0.576, thus suggesting statistically significant consistency in analysis across graders. Literature on interobserver agreement of VCDR has found moderate agreement between 6 glaucoma experts (median weighted of 0.67) while using stereoscopic conditions [11]. Compared with standard-of-care technology, D-EYE video footage suggests comparable efficacy in making CtD measurements.

## Discussion

Based on the review of 110 participants’ fundal imaging by ophthalmology residents, the pilot study indicated greater visualizability and clarity of 30-second videos of retinal imaging through the D-EYE as compared with still-image ophthalmic capture. Furthermore, the CtD measurement was more reliably measured in the 30-second video group. In addition, the likelihood of referral between the glaucomatous and healthy sample groups indicated a greater sensitivity of digital ophthalmoscopes in being able to detect retinal abnormalities, supporting the technology’s use as a screening tool. Participants noticed no change in compliance or pain associated with either of the methods used in the study.

By comparing grader assessments of the overall health of the eye (measured as a likelihood of referral) with self-reported glaucoma status by participants, the authors aimed to ascertain whether D-EYE video footage could be used to provide valuable judgments on the need for additional ophthalmic care. The use of this technology, based on the results, shows that this tool has similar value as specialized ophthalmology examinations in assessment for glaucoma. With vision loss from glaucoma being irreversible and the prevalence of glaucoma rising worldwide, implementation of this easily accessible, highly sensitive diagnostic technology could provide a multitude of health benefits while addressing any barriers to health care.

One primary limitation in the study design is the subjectivity that is associated with grading fundus images by ophthalmologists. This study attempts to limit that by operating with the input of 5 ophthalmologists grading each image acquired in an effort to reach a fair conclusion for each fundus image.

Integration of D-EYE technology into clinical settings confers advantages such as portability and steadily decreasing costs. This study has found that still images taken by the D-EYE are not yet clear enough to provide consistent optic nerve head visibility. Video footage, however, shows not only the ability to provide optic nerve head visualization, but also to assess important health metrics that can be useful both within ophthalmology clinics and in primary care settings. Future research should be aimed at assessing D-EYE efficacy in a larger population.

## Abbreviations

CtD: cup-to-disc
FDA: US Food and Drug Administration
HIPAA: Health Insurance Portability and Accountability Act
PV: proportional variability
UF: University of Florida
VCDR: vertical cup-to-disc ratio

## Data Availability

The data sets generated during and/or analyzed during this study are available from the corresponding author on reasonable request.
